# Analysis of Thick-Walled Oxygen-Free Copper Pipe Production in the Bridge Die Extrusion Process

**DOI:** 10.3390/ma18235304

**Published:** 2025-11-25

**Authors:** Marcin Knapiński, Grzegorz Banaszek, Anna Kawałek, Teresa Bajor, Grzegorz Boczkal

**Affiliations:** 1 Faculty of Production Engineering and Materials Technology, Czestochowa University of Technology, 42-201 Czestochowa, Poland; marcin.knapinski@pcz.pl (M.K.); grzegorz.banaszek@pcz.pl (G.B.); anna.kawalek@pcz.pl (A.K.); 2 Faculty of Non-Ferrous Metals, AGH University of Krakow, 30 Mickiewicza Ave, 30-059 Krakow, Poland; gboczkal@agh.edu.pl

**Keywords:** copper, pipe, extrusion, bridge die, continuous pipe extrusion process

## Abstract

This article presents the results of research on the possibility of extruding oxygen-free copper pipes in bridge dies. The possibility of continuous production of a finished product of any length with a uniformly deformed wall was analysed. One of the most important elements of the work was to determine the shape of the tool (die and bridge) that would allow durable connection of the material. Numerical studies conducted using the commercial computer programme FORGE^®^NxT 2.1, including analysis of the distribution of material temperature and hydrostatic pressure in the welding zone of the bridge die affecting the copper joint during the manufacture of tubular profiles, confirmed the validity of the research issue. The results of the numerical studies were supplemented by laboratory tests, confirming the accuracy of the selected variant of the finished product manufacturing process. The process of bonding under conditions of two-part material compression was used for physical modelling of copper welding. The tests were conducted using the Gleeble 3800 metallurgical process simulator with the PocketJaw module. Based on the analysis of the obtained results, it was found that for tubes with a wall-thickness-to-inner-diameter ratio of 0.5, it is justified to use tools with a longer sizing section and welding chamber, as well as a larger mandrel generating-line angle within the welding chamber.

## 1. Introduction

Copper pipes have been well-regarded in many industries for decades. Particularly noteworthy are the energy and construction sectors, where copper pipes are used in photovoltaic, water supply, heating, and gas installations [1,2,3]. Pure copper, compared to pure aluminium, is a relatively expensive material and is also more difficult to deform, but it is characterised by high durability, which is an unquestionable advantage. Furthermore, copper is a material characterised by high mechanical strength and long service life, and products made of it are resistant to damage, high temperatures, and, most importantly, corrosion. All these features mean that installations made of copper pipes, for instance, operate reliably for many years, which is a cost-effective and safe investment in the long term. A feature of installations made of Cu is that there is no need for special protective coatings, as the material is covered with a patina, which acts as a natural protective barrier. All these advantages make copper installations less susceptible to leaks and damage [4,5,6].

The use of long-life-cycle products in the context of contemporary challenges faced by companies is justified from the point of view of ecology [7] and the economics of production process planning. When comparing PVC (polyvinyl chloride) pipes used for water transmission with copper pipes, research shows that the use of copper solutions is more beneficial for users [8].

The wide range of applications for products made of Cu alloys requires a search for plastic forming processes that use low-emission and environmentally friendly solutions, generating small amounts of post-production waste. All these elements pose a challenge for the manufacturing industry, which is obliged to produce components with high strength, fatigue resistance, and thermal corrosion resistance. Extrusion is a process that, in addition to the above advantages, also allows for greater utilisation of raw materials. It is a highly efficient method with low energy consumption. A properly designed and performed extrusion process allows for the production of products with good mechanical properties [9,10,11,12,13,14,15].

A wide variety of shapes of manufactured structural elements can be achieved by using the bridge-chamber die extrusion process. This type of solution allows for a reduction in process parameters such as temperature or pressure [12,16,17,18,19].

In [20,21], the results of experimental research on the production of round copper rods in the hot extrusion process were presented. The results confirm that the manufactured product shows very good mechanical properties and a homogeneous structure throughout its cross-section.

The authors of the work conducted research on unconventional plastic forming processes, including the production of oxygen-free copper pipes with a wall thickness to internal diameter ratio of 0.25, using the bridge-chamber die extrusion method [12]. Numerical studies allowed us to determine the minimum charge temperature required to ensure complete copper welding in the die welding chamber, which is 600 °C, as confirmed by physical tests.

The aim of this study was to determine the influence of selected tool-shape parameters, such as the angle of the mandrel in the welding chamber area, the length of the welding chamber, and the length of the calibrating part of the pipe forming die, on the distribution of temperature and hydrostatic pressure in the material inside the bridge die It has also been demonstrated that the thermal and mechanical conditions occurring in copper during the extrusion of a round tube with a wall thickness to internal diameter ratio of 0.5 are conducive to the formation of a proper bond between the material separated by the die bridge.

## 2. Material and Research Methodology

In order to achieve the objective of the study, research was conducted on the welding of oxygen-free copper using physical modelling of the pressure welding process under conditions of cylindrical sample swelling and numerical modelling, including an analysis of the stresses and temperatures occurring in the welding and extrusion area under continuous conditions for a copper pipe with a wall thickness to internal diameter ratio of 0.5.

### 2.1. Methodology for Copper Weld Quality Analysis Under Physical Simulation Conditions

**Figure 1:**
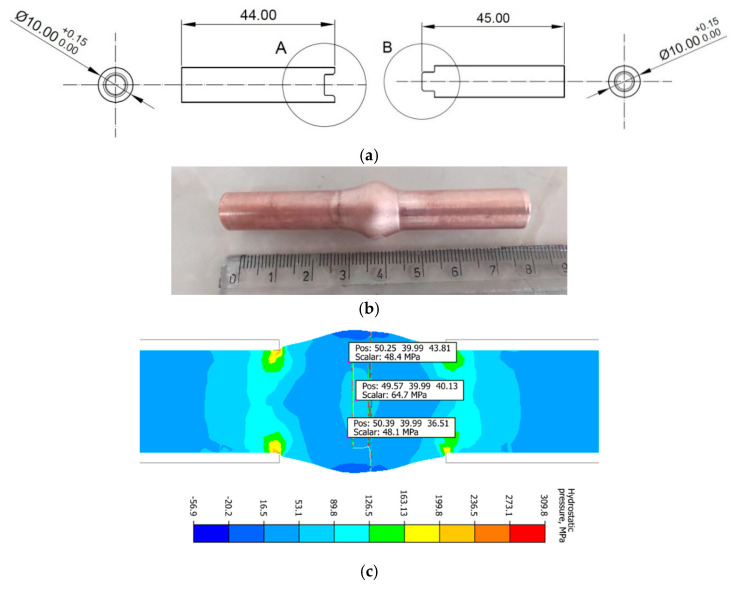
Sample used for physical simulations of the copper welding process [12]. (**a**) Sample dimensions, (**b**) view of the joined sample after bulging, (**c**) distribution of values of hydrostatic pressure [MPa] in the sample during bulging.

An important issue that arises during the production of pipe profiles under extrusion conditions using complex bridge dies is the knowledge of thermal and mechanical conditions that guarantee a high-quality connection of the material that has been initially divided by the bridge die. The authors presented a methodology for physical simulation of copper pressure welding in [12]. The samples, consisting of two parts, were made according to Figure 1a and pressed using a Gleeble 3800 metallurgical process simulator with a PocketJaw module (DSI, Poestenkill, NY, USA) in a configuration normally used for SICO (strain-induced crack opening) tests [12,22,23,24]. The view of the sample after bulging is shown in Figure 1b. Furthermore, in [12], a numerical simulation of the swelling process was presented in order to determine the temperature and hydrostatic pressure distributions (Figure 1c) occurring in the copper joint area. Based on the analysis of the obtained test results, it was found that under physical simulation conditions, when the total length of the sample is reduced from 86 mm by 10 mm, hydrostatic pressure values in the weld area range from 45 to 65 MPa, with maximum values occurring in the sample axis. In [12], the quality of the weld was assessed based on observations using a Nikon Eclipse MA200 optical microscope (Nikon Industrial Metrology, Tokyo, Japan). Based on the observations made, it was found that at a temperature of 520 °C, no welding was achieved, at temperatures of 570 °C and 600 °C, partial welding was achieved, and at temperatures of 640 °C and above, complete welding of the material was achieved.

**Figure 2:**
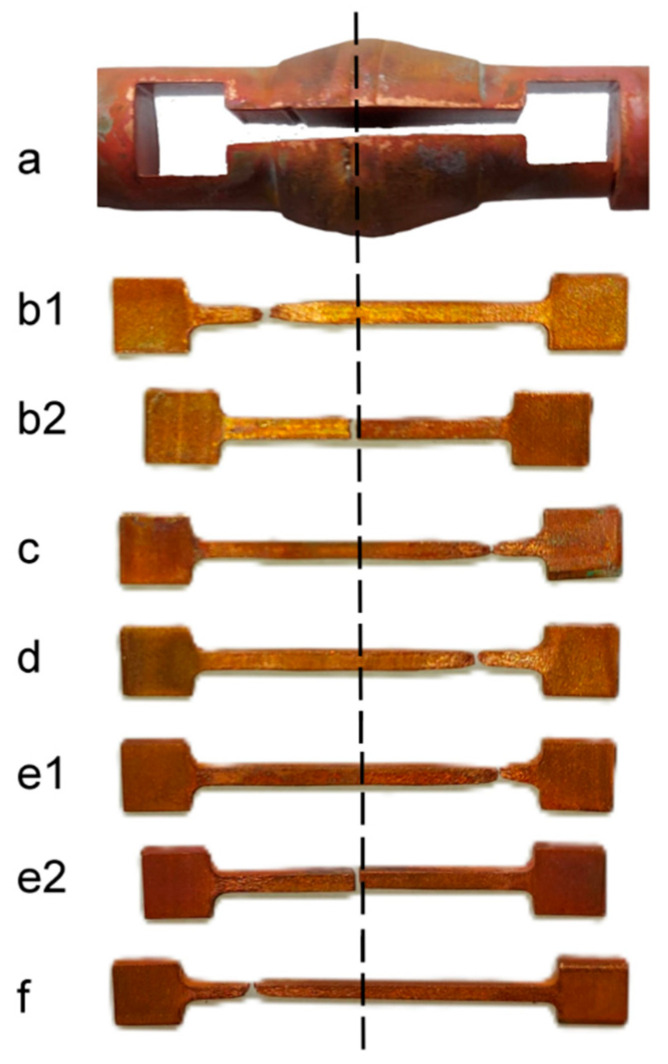
View of samples after pressure welding; the dotted line marks the welding plane. (**a**) Method of sampling for tensile testing, (**b1**) 540 °C axis of the welded sample, (**b2**) 540 °C edge of the welded sample, (**c**) 570 °C axis of the welded sample, (**d**) 600 °C axis of the welded sample, (**e1**) 640 °C axis of the welded sample, (**e2**) 640 °C edge of the welded sample, (**f**) reference sample.

In order to confirm the quality of the copper connection obtained in the SICO sample under the tested conditions, the authors conducted physical simulations of the welding process at temperatures of 540 °C, 570 °C, 600 °C, and 640 °C, and then micro-samples were taken from the samples obtained after plastic deformation for tensile testing (Figure 2). The dimensions of the micro-samples were a thickness and a width of the working part of approximately 1.6 mm and a base length of 21 mm. The method of cutting micro-samples, together with examples after breaking in the tensile test, is shown in Figure 2. Five micro-samples were cut from each sample subjected to bulging at a specific temperature. The reference sample (Figure 2f) was taken from a solid material that had not been deformed to determine whether the material’s mechanical properties changed during deformation and welding. The method for collecting micro-samples from the reference sample was the same as for the other micro-samples taken from the material after plastic deformation.

Tensile tests were performed using a TM-SM Instron testing machine with a modified measuring track and a 5 KN head (Norwood, MA, USA) at an average traverse speed of 0.02 mm/s and an ambient temperature of 20 °C.

### 2.2. Methodology for Numerical Modelling of the Pipe Extrusion Process

The analysis of the pipe extrusion process using a separating bridge and a calibrating die was performed using commercial FORGE^®^NxT 2.1 software, based on the finite element method (FEM). A cylindrical model sample with a diameter of 20 mm and a length of 18 mm, made of pure copper (Cu), was used for the tests [25].

The software used enables thermomechanical simulation of plastic working processes. Detailed information on the temperature, energy, stress–strain, and thermomechanical and friction functions used in the calculations was described in [12]. The rheological data of oxygen-free copper for the FORGE^®^NxT 2.1 software were introduced in a tabular form based on experimental results obtained under real deformation process conditions. Providing the data in this format to numerical simulation programmes enables higher calculation accuracy over a wide range of deformation temperatures of the material.

In this analysis, a thermo-viscoplastic model of a deformed body was adopted, based on the theory of large plastic deformations. The finite element mesh was created from tetrahedral elements. In the batch model, the number of nodes was 30,001, and the number of elements was 151,726.

The following boundary conditions were assumed:

- A coefficient of friction between the tool surfaces and the material of μ = 0.3,
- A coefficient of heat transfer between tools and material of λ = 8100 W/m^2^ K,
- A coefficient of heat transfer between Cu alloy and the environment of α = 7 W/m^2^ K.

The initial conditions of the extrusion process included the following:

- A piston feed speed of v = 10 mm/s,
- An initial charge temperature of 600 °C,
- An ambient temperature of 25 °C,
- A tool temperature of 350 °C,
- The length of the calibrating part of the die L_k_ was varied in the range of 1–4 mm,
- The length of the welding chamber L_kz_ was varied within the range of 2–3 mm,
- The angle of the mandrel in the welding chamber α was varied within the range of 21–41°.

**Figure 3:**
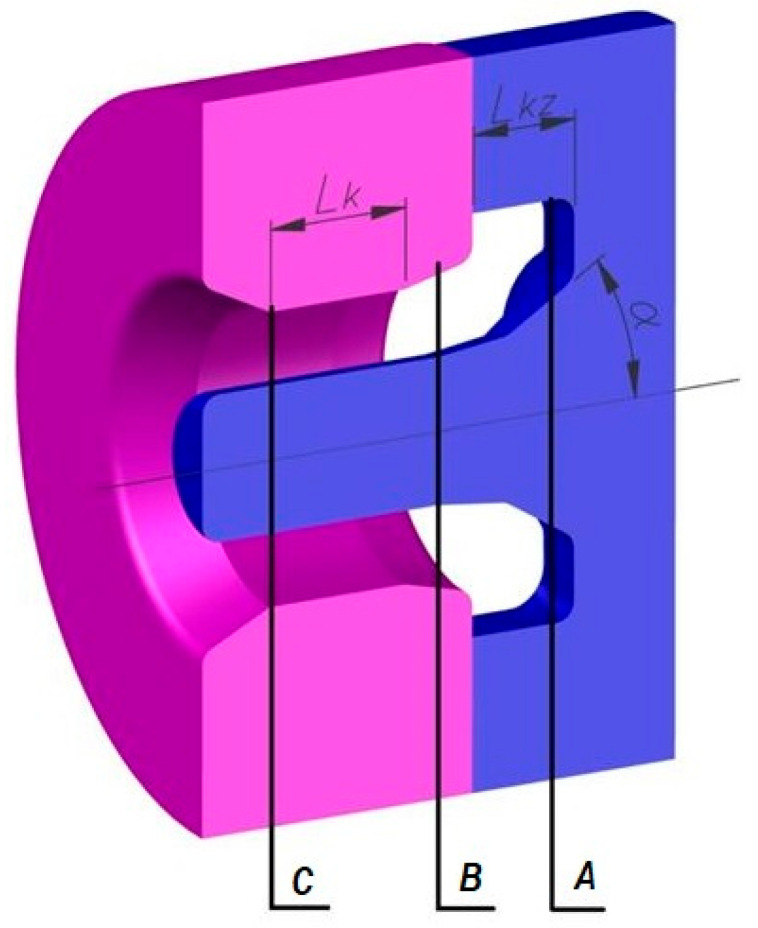
Cross-section of the die consisting of a separating and a calibrating part.

Figure 3 presents the cross-sections (A), (B), and (C), perpendicular to the material axis, located in characteristic areas of the extrusion process: the beginning of the welding chamber (A), the middle of the deformation zone in the die (B), and the die exit (C). For the indicated cross-sections, an analysis of the temperature and hydrostatic pressure distribution during the tube extrusion process was carried out using a die consisting of a separating bridge and a calibrating section.

## 3. Results and Analysis of Model Tests

### 3.1. Results of Tensile Tests of Copper After Bulging Process

**Figure 4:**
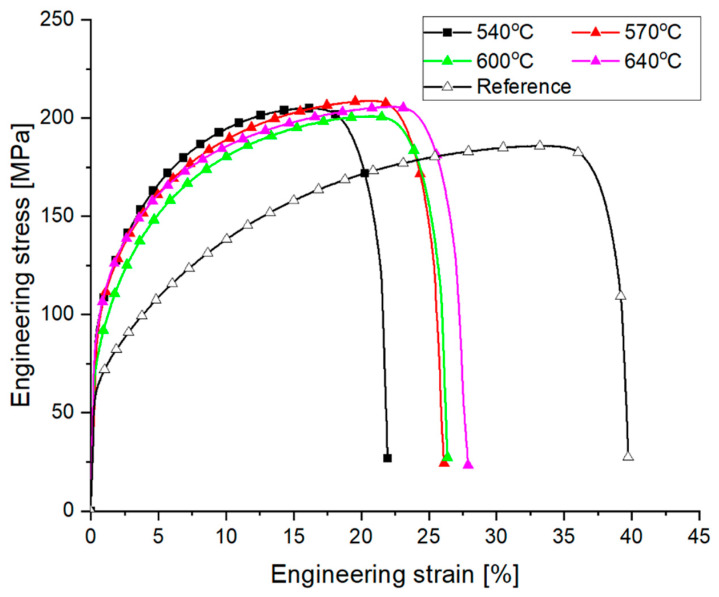
Selected tensile curves of micro-samples cut from the axis of the welded material during plastic deformation at different temperatures.

Micro-samples taken from the base copper specimen after welding under bulging conditions at various temperatures were subjected to strength tests to illustrate the behaviour of the material in different states, depending on the location from which each micro-sample was cut. A characteristic feature of all the collected samples was the position of the weld along the length of the micro-sample, which is shown by the dashed line in Figure 2. However, the micro-samples were taken from the axis of the bulged material as well as from areas located closer to the surface; therefore, the weld was formed under different values of hydrostatic pressure. Figure 4 presents the tensile curves of micro-samples taken from the axial part of the copper after bulging, as well as the tensile curve of the solid material that was not subjected to the bulging process.

When observing the shape of the tensile curves of the micro-samples after bulging, it can be noted that the deformation process conducted within the temperature range of 540–640 °C caused material strengthening, which is evident from an increase in strength of approximately 20 MPa compared to the reference material. At the same time, a decrease in ductility is observed, as the elongation (A_gt_), i.e., the percentage increase in sample length to reach the maximum force, decreased compared to the reference material by approximately 15% for a bulging temperature of 640 °C and by approximately 17% for a bulging temperature of 540 °C.

### 3.2. Analysis of Temperature Distribution in Numerical Modelling of the Extrusion Process

**Figure 5:**
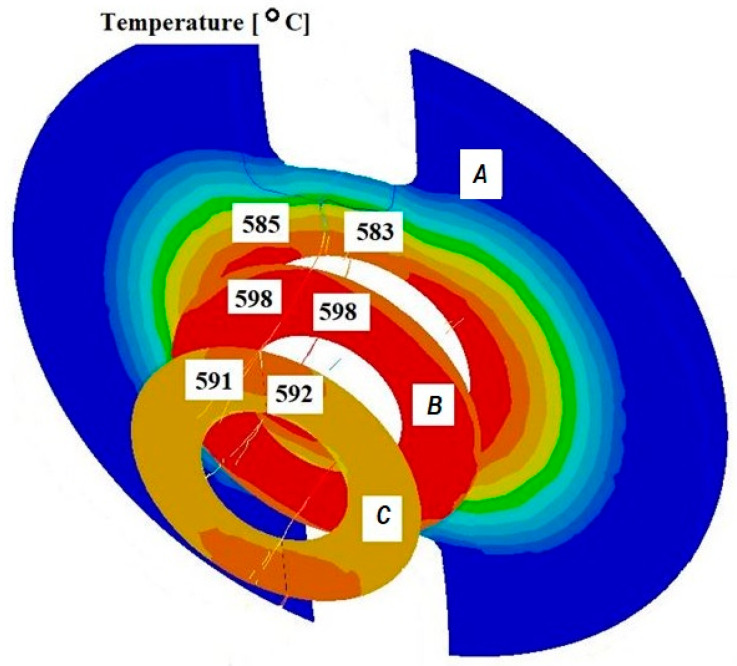
Distribution of values of temperature in the cross-sections of the welded pipe, with a calibrating die section length of 1 mm, a welding chamber length of 2 mm, and a mandrel apex angle in the welding chamber of 32°.

**Figure 6:**
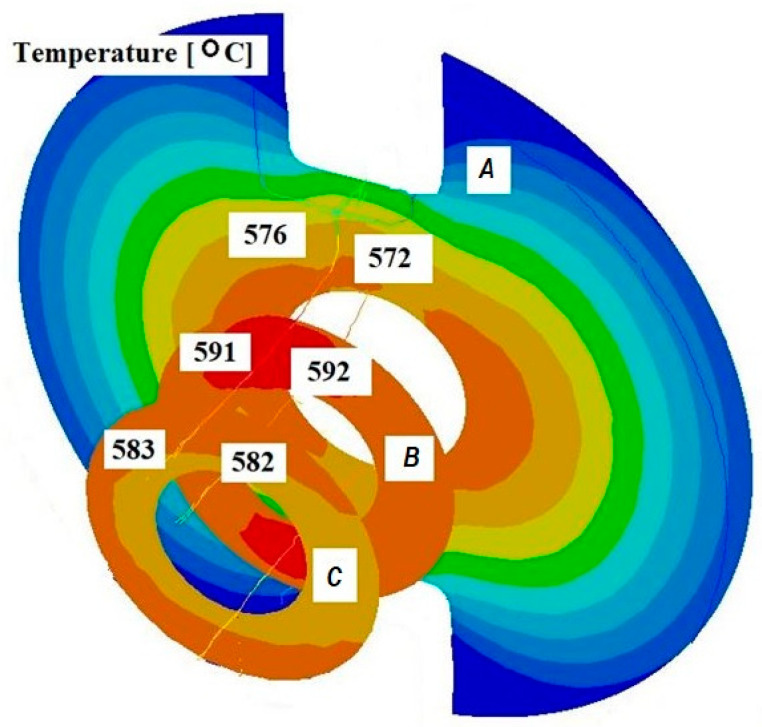
Distribution of values of temperature in the cross-sections of the welded pipe, with a calibrating die section length of 4 mm, a welding chamber length of 3 mm, and a mandrel apex angle in the welding chamber of 32°.

Figure 5 and Figure 6 present selected temperature distributions obtained from the simulation of the extrusion process of oxygen-free copper tubes using a separating bridge and a calibrating die for an initial batch temperature of 600 °C. The lengths of the calibrating sections were 1 and 4 mm, and the lengths of the welding chambers were 2 and 3 mm.

Based on the analysis of the results of the pipe extrusion process modelling, it was found that, regardless of changes in the geometric parameters of the tools, similar temperature values were obtained in the examined cross-sections. This means that, within the analysed range, changes in the geometric parameters did not have a significant impact on the temperature distribution.

Based on the conducted analysis of the numerical modelling results, it can be concluded that the temperature maintained during the process is sufficient for proper welding of the material.

### 3.3. Analysis of the Hydrostatic Pressure Distribution and Material Flow Velocity in Numerical Modelling of the Extrusion Process

**Figure 7:**
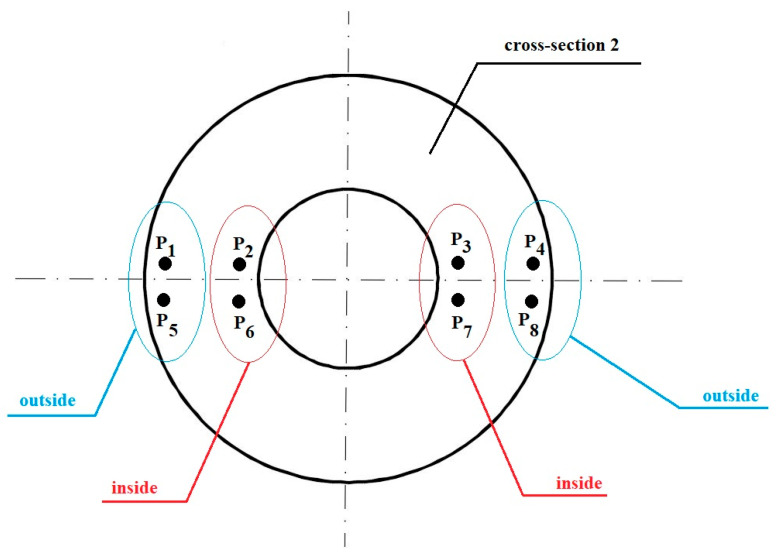
Position of the characteristic points from which the hydrostatic pressure values were recorded.

The analysis of the hydrostatic pressure distribution in the welding and extrusion zone during numerical modelling of the process began with examining the influence of selected factors on the average pressure values in characteristic areas of the material cross-section. It was located at the midpoint of the crushing section of the die. In the selected plane, the material joined in the welding chamber is already shaped into a tube and undergoes compression to achieve the desired product dimensions. Therefore, this is the area where maintaining the compression state and the highest possible hydrostatic pressure values in the copper is particularly important. Furthermore, it is important that the pressures in the formed pipe wall do not differ significantly between the part shaped by the mandrel and the part directly affected by the die. For this reason, eight characteristic points were selected on the plane, located on both sides of the weld in the layers near the outer and inner surfaces (Figure 7). Then, the average values of hydrostatic pressure were calculated from four points in the outer layers and four points in the inner layers.

**Figure 8:**
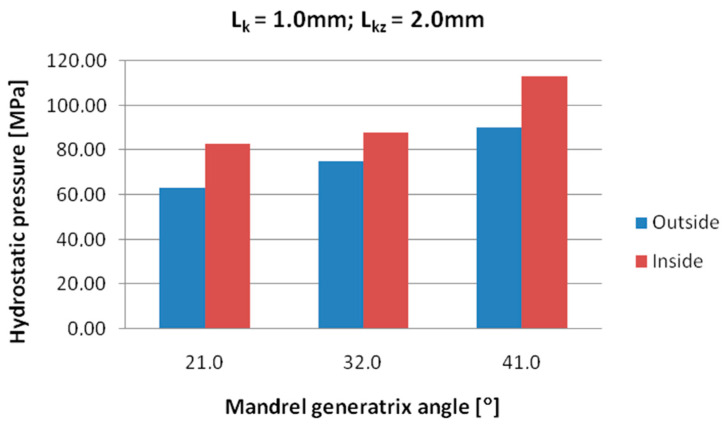
Dependence of the average hydrostatic pressure on the mandrel apex angle in the areas shown in Figure 7.

**Figure 9:**
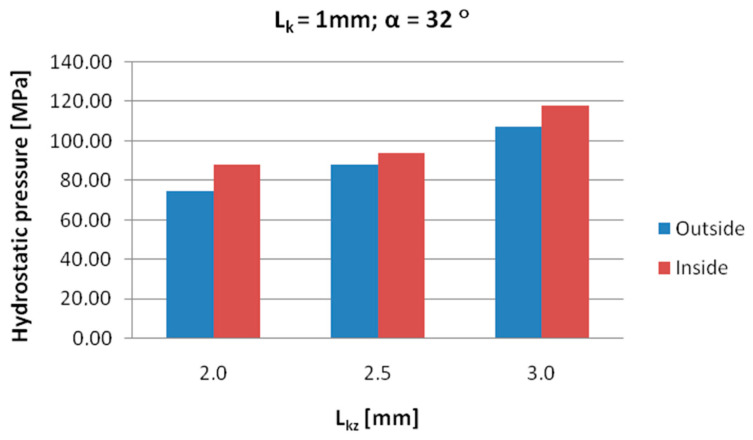
Dependence of the average hydrostatic pressure on the length of the welding chamber in the areas shown in Figure 7.

**Figure 10:**
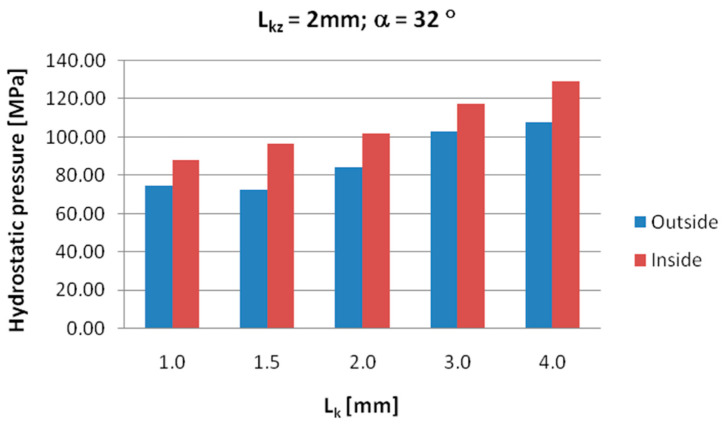
Dependence of the average hydrostatic pressure on the length of the calibrating die section in the areas shown in Figure 7.

**Figure 11:**
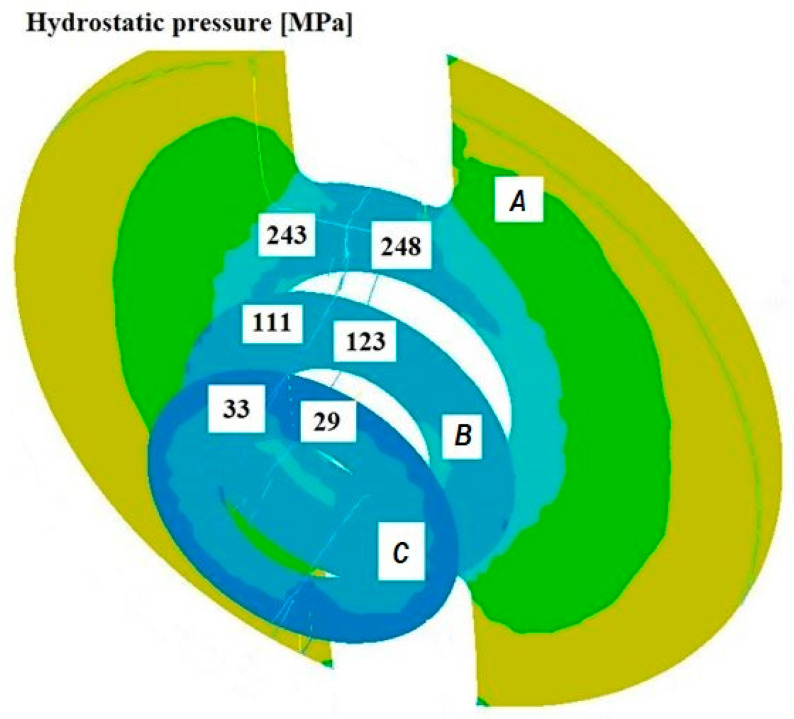
Distribution of values of hydrostatic pressure in the cross-sections of the welded pipe, with a calibrating die section length of 1 mm, a welding chamber length of 2 mm, and a mandrel apex angle in the welding chamber of 41°.

**Figure 12:**
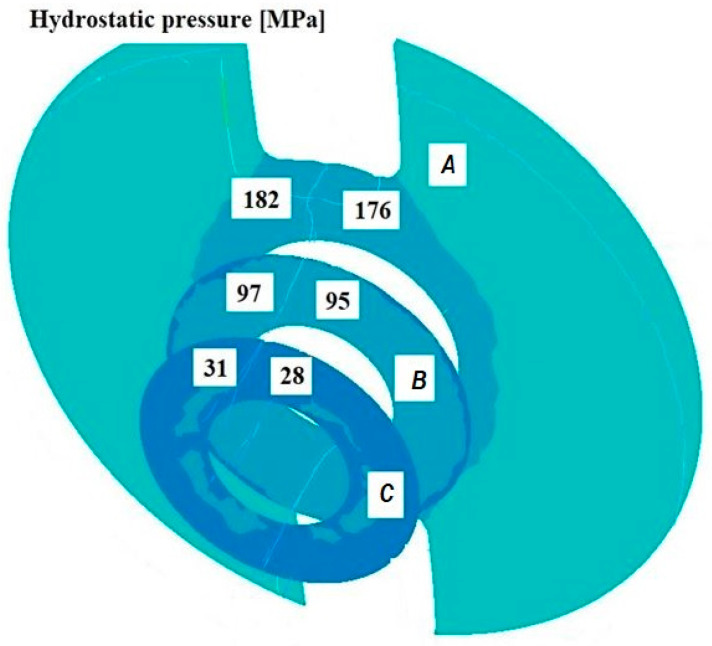
Distribution of values of hydrostatic pressure in the cross-sections of the welded pipe, with a calibrating die section length of 1 mm, a welding chamber length of 2 mm, and a mandrel apex angle in the welding chamber of 32°.

**Figure 13:**
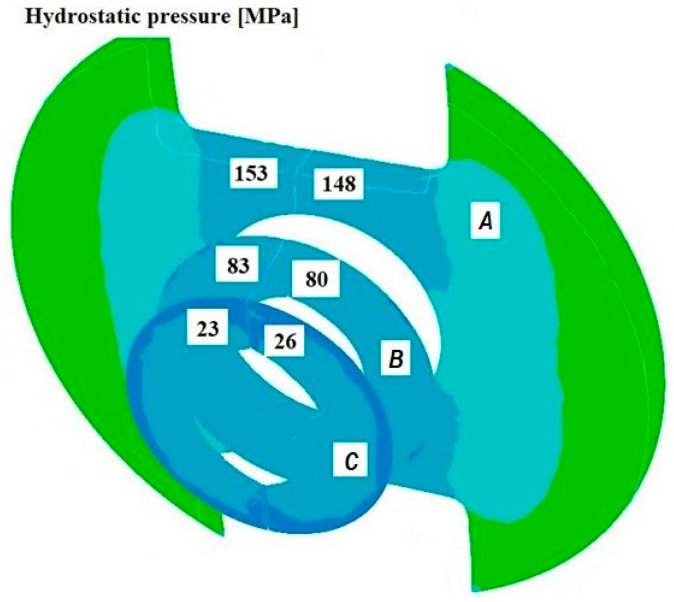
Distribution of values of hydrostatic pressure in the cross-sections of the welded pipe, with a calibrating die section length of 1 mm, a welding chamber length of 2 mm, and a mandrel apex angle in the welding chamber of 21°.

**Figure 14:**
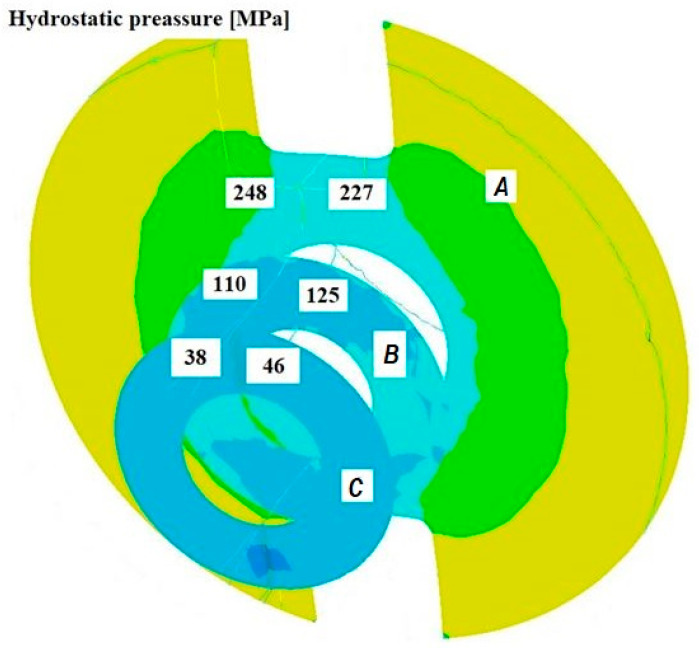
Distribution of values of hydrostatic pressure in the cross-sections of the welded pipe, with a calibrating die section length of 1 mm, a welding chamber length of 3 mm, and a mandrel apex angle in the welding chamber of 32°.

**Figure 15:**
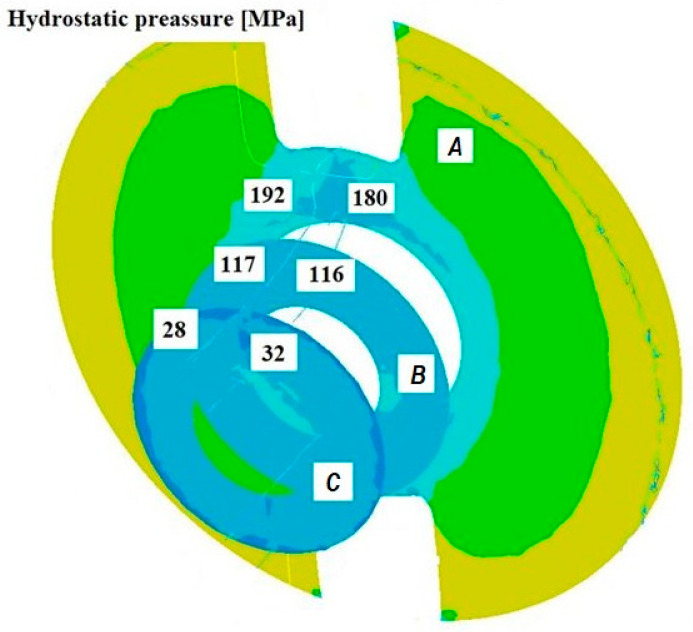
Distribution of values of hydrostatic pressure in the cross-sections of the welded pipe, with a calibrating die section length of 4 mm, a welding chamber length of 2 mm, and a mandrel apex angle in the welding chamber of 32°.

**Figure 16:**
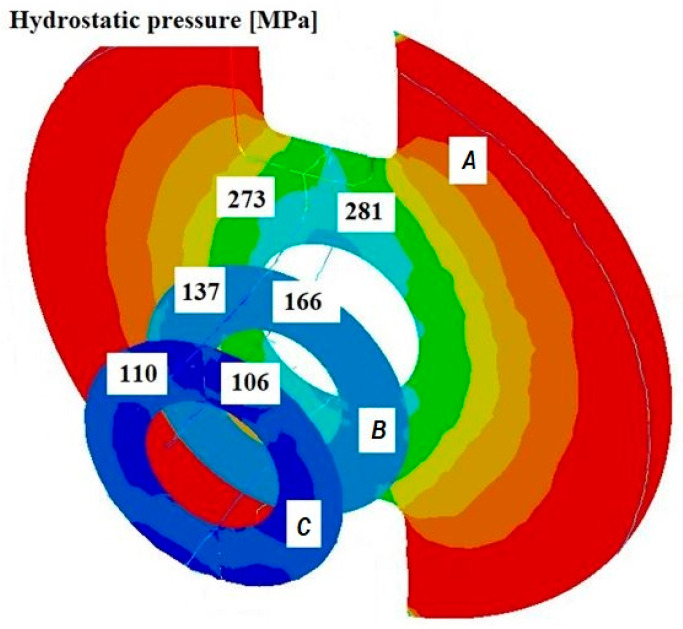
Distribution of values of hydrostatic pressure in the cross-sections of the welded pipe, with a calibrating die section length of 4 mm, a welding chamber length of 3 mm, and a mandrel apex angle in the welding chamber of 32°.

Figure 8, Figure 9 and Figure 10 show the relationships of the average hydrostatic pressure values in the inner and outer layers of the formed pipe with the mandrel apex angle α, the length of the welding chamber, and the length of the calibrating zone. Figure 11, Figure 12, Figure 13, Figure 14, Figure 15 and Figure 16, on the other hand, present example distributions of hydrostatic pressure values in three cross-sections perpendicular to the axis of the extruded material, as marked in Figure 3.

Data presented in Figure 8 indicate that higher hydrostatic pressure values were obtained in the zone near the inner diameter of the welded pipe for all investigated mandrel apex angles. As the value of this angle increased, the hydrostatic pressure also increased, reaching maximum values at an angle of 41°. A differentiation of hydrostatic pressure values between the outer and inner areas of the material wall cross-section is also observed.

Based on the data shown in Figure 9, it can be stated that the longer the welding chamber, the longer the material interacts with the tool surface, which results in higher frictional resistance and higher hydrostatic pressure values in the zone around the inner diameter of the welded pipe. A shorter welding chamber causes faster material flow and lower hydrostatic pressure values because the zone of intense compression is smaller.

Data presented in Figure 10 indicate that extending the calibrating section increases the resistance to material flow. As a result, the length of the deformation zone increases, since the material undergoes more intensive compression and friction along the die walls. This leads to an increase in hydrostatic pressure values in the areas around the inner diameter of the pipe.

Figure 11, Figure 12, Figure 13, Figure 14, Figure 15 and Figure 16 illustrate example distributions of hydrostatic pressure values in the die cross-sections. For these examples, a constant length of the calibrating section equal to 1 mm and a welding chamber length of 2 mm were assumed. The parameter subject to variation was the mandrel apex angle in the welding chamber, with values in the range of 21–41°.

When analysing the data in Figure 11, Figure 12 and Figure 13, it can be stated that the highest hydrostatic pressure values occur in the welding zone of the die (cross-section A) at the maximum mandrel apex angle α of 41°, with the other process parameters held constant. At this angle, the material flows more intensively into the welding chamber, and there is a higher concentration of compressive stresses, which leads to higher hydrostatic pressure values.

The research results presented in Figure 14 and Figure 16 confirm that the length of the calibrating section of the die has a significant impact on the formation of the deformation zone and the hydrostatic pressure values in the extrusion process. A short calibrating section may not provide adequate conditions for material welding, whereas a long section increases the resistance to material flow, leading to higher extrusion forces and hydrostatic pressure in the welding chamber and in the material subjected to the action of the die crushing section. This positively affects the welding conditions but may reduce extrusion efficiency.

Based on the results shown in Figure 14 and Figure 15, it can be concluded that extending the welding chamber promotes favourable conditions for material welding (higher hydrostatic pressure), but at the same time increases frictional forces, thereby increasing the resistance to plastic flow of the material in this zone. A deformation zone that is too short may lead to a decrease in hydrostatic pressure values and, in consequence, prevent proper material welding.

## 4. Discussion of the Obtained Modelling Results

### 4.1. Discussion of the Physical Simulation Results of the Welding Process

**Figure 17:**
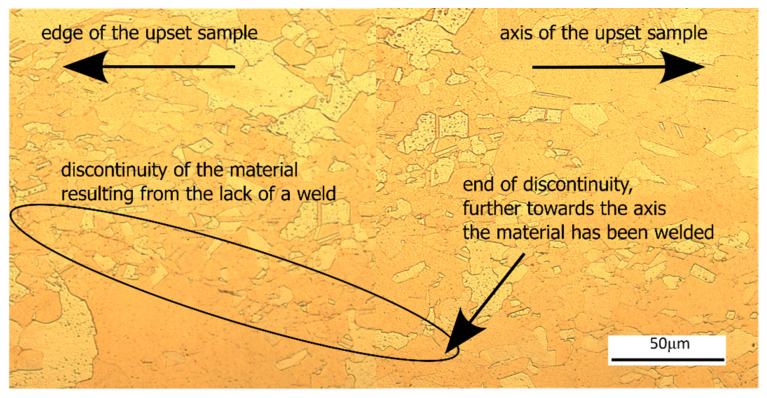
Example images of the bonding zone after the upsetting process at 540 °C.

When considering only the obtained tensile curves of copper after the pressure welding process, it can be preliminarily assumed that an acceptable material weld was achieved in all investigated cases. However, the results of microstructural observations presented in Figure 17 indicate that at temperatures of 540 °C, a complete copper bond was not achieved, with a visible grain boundary, particularly in the layers closer to the surface of the sample.

**Figure 18:**
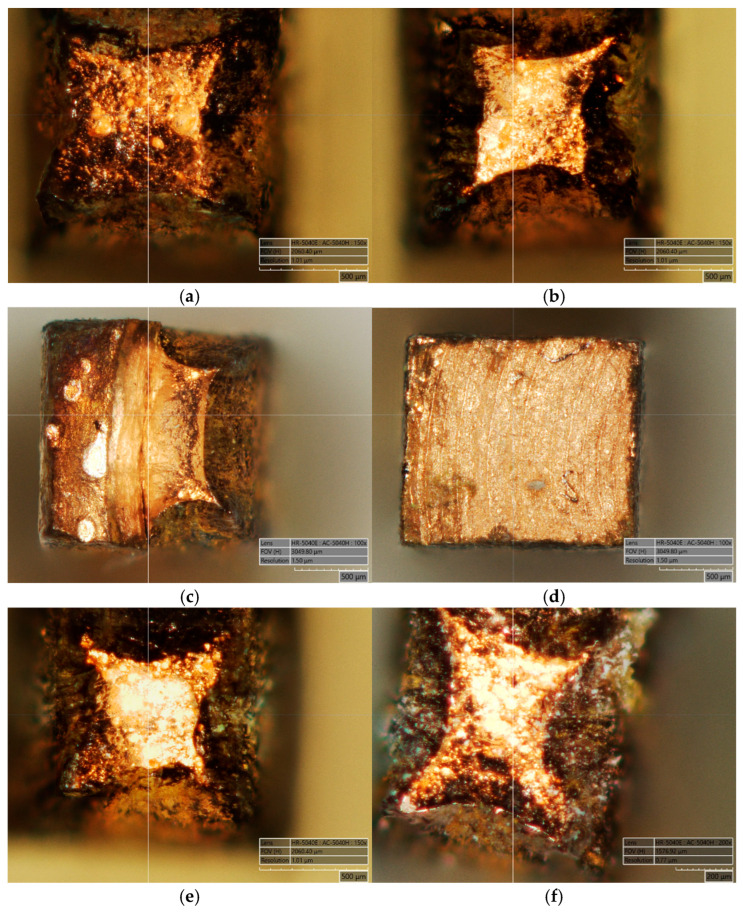
Views of the fracture surfaces of micro-samples after tensile tests, as shown in Figure 2: (**a**) b1—axis of the bulged sample at 540 °C, (**b**) e1—axis of the bulged sample at 640 °C, (**c**) b2—edge of the bulged sample at 540 °C, (**d**) e2—edge of the forged sample at 640 °C, (**e**) c—axis of the bulged sample at 570 °C, (**f**) f—axis of the reference sample.

Explanation of this phenomenon requires a comprehensive analysis of all research results, in particular the examination of the fracture locations of the samples during tensile tests and the distribution of thermomechanical conditions in the sample cross-sections during welding. Figure 18 shows examples of micro-samples after the tensile test, with the welding zone indicated. The fractures shown in this figure are of diverse nature. In the case of micro-samples taken from the sample axis after the upsetting operation (Figure 18a,b,e) and from the solid material without deformation (Figure 18f), typical ductile fractures are visible. Furthermore, it can be seen that the material fractured along grain boundaries, with individual grains of varying sizes and rounded surfaces clearly visible. A different state can be observed in the fractures formed in micro-samples taken from areas near the surface of the upset samples. Of particular interest is the fracture shown in Figure 18c, which shows a ductile fracture (in the right part of the fracture) and delamination of the material that was not properly welded (in the left part of the fracture). In contrast, the fracture shown in Figure 18d does not exhibit visible features of a ductile fracture. It was created during the tension of a micro-sample taken from an area of material that was only point-joined due to insufficient pressure at the interface between the joined surfaces. It also shows the residual macrostructure of the sample surface prepared for joining by subtractive mechanical processing (characteristic arc-shaped stripes).

It can be seen from this figure that micro-samples taken from the axis of the forged material (b1), (c), (d), and (e1) fractured in the solid material zone, regardless of the welding temperature. In contrast, samples taken near the edge fractured in the joint area, for example, (b2) and (e2). When relating these results to the distribution of hydrostatic pressure in the deformation zone during sample forging, it can be observed (Figure 1c) that pressures of approximately 65 MPa occurred along the material axis, while in the edge layers, the hydrostatic pressure ranged from about 16 to 53 MPa. Moreover, in the edge layers after deformation, areas could form in the welding plane consisting of surfaces parallel to the sample axis (Figure 1a—details A and B), whose initial positions prevented the attainment of thermomechanical conditions sufficient for proper copper bonding.

To confirm the phenomena described above, Figure 18 presents images of various fracture surfaces obtained in the tensile tests using an HRX-01 confocal microscope (HIROX, Limonest, France). When analysing the obtained data, it can be concluded that within the studied temperature range, a complete copper bond was achieved along the axis of the forged samples. Micro-samples taken from the axis of the bulged samples fractured in the solid material, outside the joint area, with representative fracture surfaces shown in Figure 18a,b,e. These are ductile fractures, typical for copper, where the crack propagated along the grain boundaries. A similar fracture was observed for the solid material that was not subjected to plastic deformation (Figure 18f). Figure 18c,d show the fractures of micro-samples taken from the lateral layer of the bulged sample at 540 °C and 640 °C, respectively. For the micro-sample shown in Figure 18c, partial bonding of the material was observed, whereas for the sample shown in Figure 18d, bonding occurred only at discrete points. The cause is the previously mentioned initial shape of the samples used for the welding simulation shown in Figure 1a. The lateral surfaces lying outside the groove area, as well as the surfaces parallel to the sample axis, undergo deformation during forging; however, their arrangement prevents material bonding in this region.

The analysis of thermomechanical conditions during pressure welding of copper conducted in this study showed that within the investigated temperature range, i.e., from 540 °C and above, oxygen-free copper achieves permanent bonding if the hydrostatic pressure in the welding zone exceeds 55 MPa.

### 4.2. Discussion of the Numerical Simulation Results of the Welding Process

In all analysed cases, the maximum stress values were located in the welding chamber area. This phenomenon is beneficial for the welding process because higher hydrostatic pressure promotes effective material bonding. Results of physical tests conducted using the Gleeble 3800 metallurgical process simulator indicate that welding at the assumed initial temperature of the model rod occurred at hydrostatic pressures exceeding 55 MPa. In the case of numerical simulations, the obtained hydrostatic pressure values were significantly higher than those in the experimental tests.

During the physical modelling of the welding process of copper samples (described in Ref. [12]) at a temperature of 600 °C, the maximum upsetting force reached 1800 kG, whereas in the numerical simulations conducted under the same conditions, the force was 1550 kG, which corresponds to a hydrostatic pressure in the range of 45–65 MPa. The difference between these results was approximately 14%.

An additional analysis of the weld quality obtained in the physical experiment showed that at 600 °C, the hydrostatic pressure occurring in the axial part of the sample at approximately 65 MPa is sufficient to achieve a fully bonded joint. In the numerical simulations of the tube extrusion process in bridge dies, the hydrostatic pressure values ranged from 80 to 163 MPa, indicating that in every analysed case, the hydrostatic pressure exceeded the value required to ensure complete welding of the material.

However, it is important to note that the analysis of the average hydrostatic pressure values in the wall layers of the forming pipe, both on the mandrel side and the die side, revealed differences. This affects the material flow after exiting the die and simultaneously causes a variation in mechanical properties along the wall thickness. To explain the reasons for this phenomenon, an analysis was conducted of the distributions of hydrostatic pressure and material flow velocity in a longitudinal section located in the theoretical zone of the copper weld, which had been initially separated by the die bridge.

**Figure 19:**
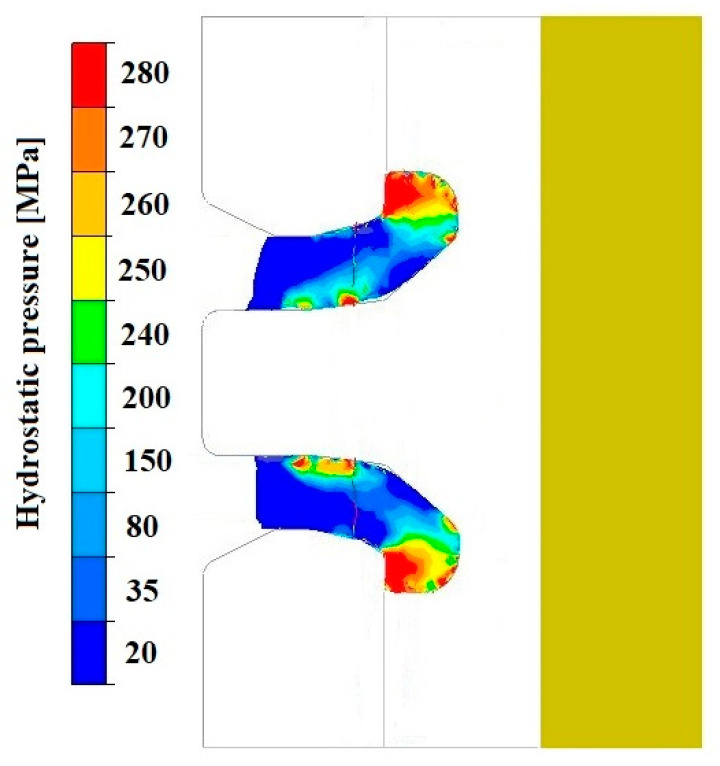
Distribution of values of hydrostatic pressure in the longitudinal section of the welded pipe, with a calibrating die section length of 1 mm, a welding chamber length of 2 mm, and a mandrel apex angle in the welding chamber of 41°.

**Figure 20:**
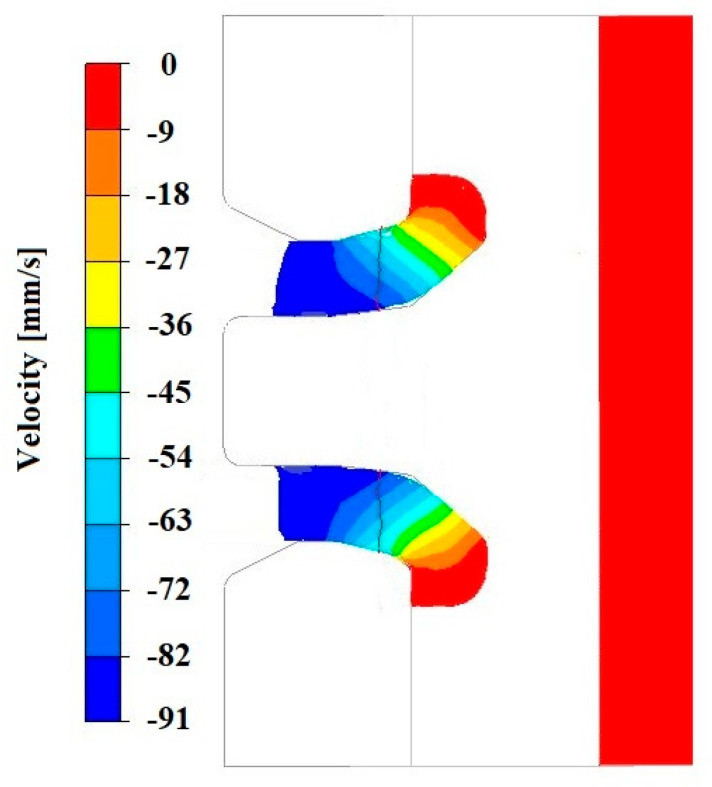
Distribution of values of material flow velocity in the longitudinal section of the welded pipe, with a calibrating die section length of 1 mm, a welding chamber length of 2 mm, and a mandrel apex angle in the welding chamber of 41°.

**Figure 21:**
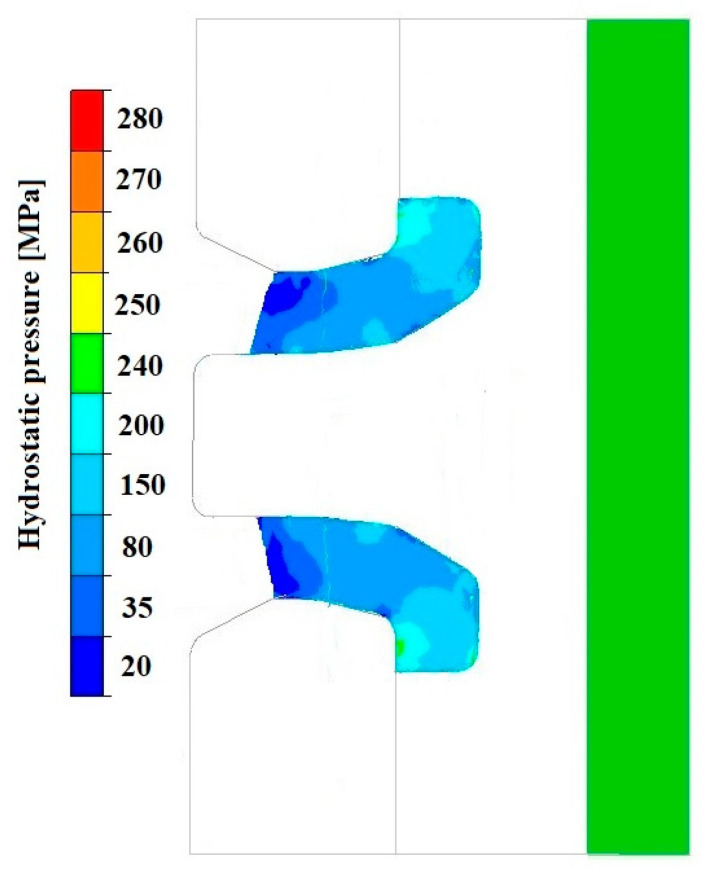
Distribution of values of hydrostatic pressure in the longitudinal section of the welded pipe, with a calibrating die section length of 1 mm, a welding chamber length of 2 mm, and a mandrel apex angle in the welding chamber of 32°.

**Figure 22:**
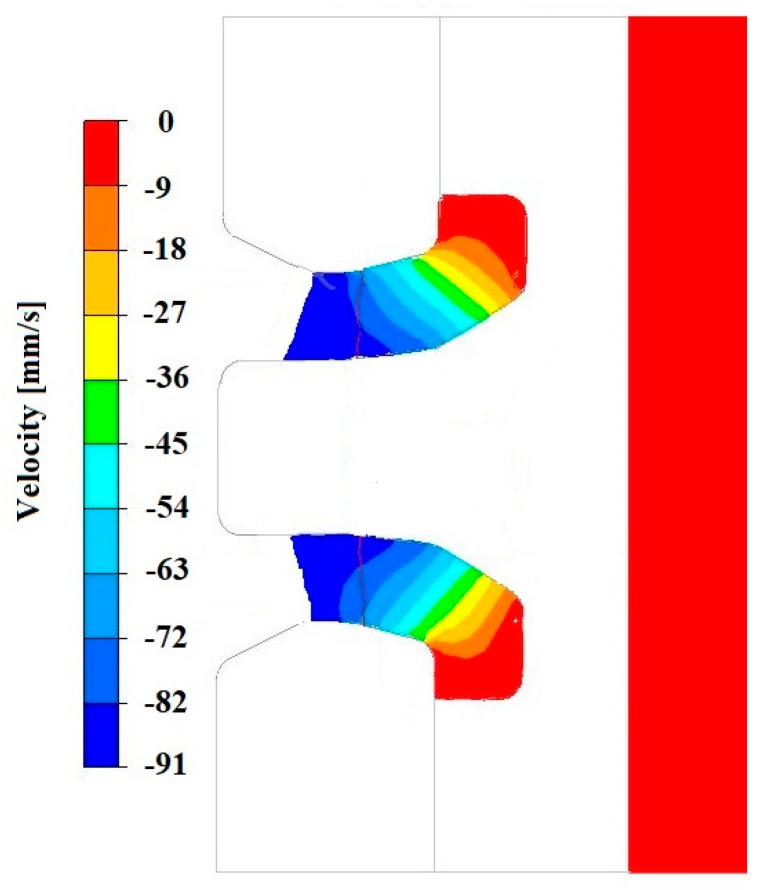
Distribution of values of material flow velocity in the longitudinal section of the welded pipe, with a calibrating die section length of 1 mm, a welding chamber length of 2 mm, and a mandrel apex angle in the welding chamber of 32°.

**Figure 23:**
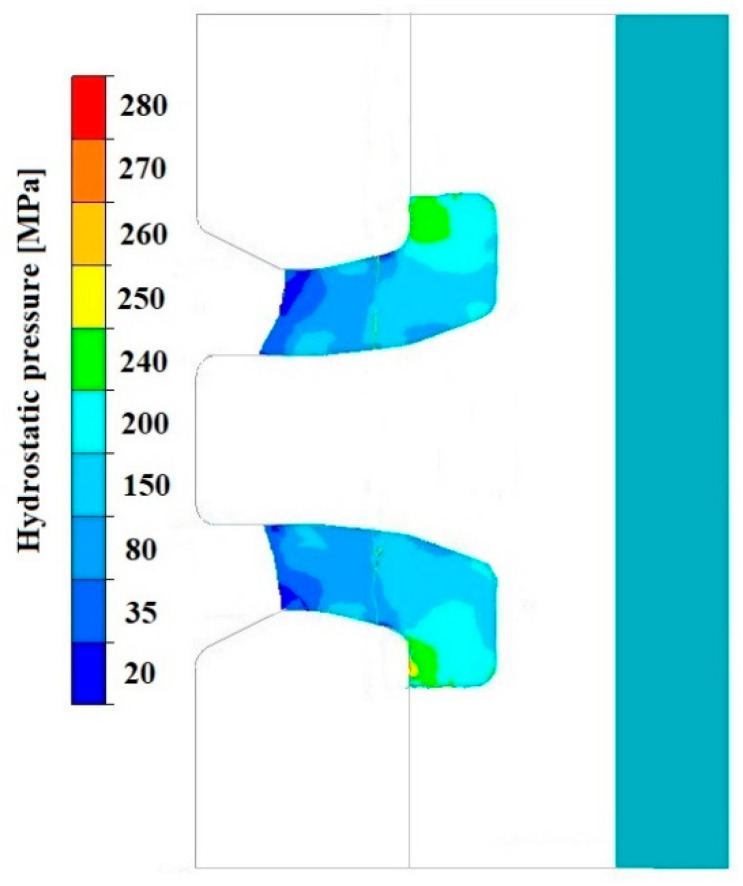
Distribution of values of hydrostatic pressure in the longitudinal section of the welded pipe, with a calibrating die section length of 1 mm, a welding chamber length of 2 mm, and a mandrel apex angle in the welding chamber of 21°.

**Figure 24:**
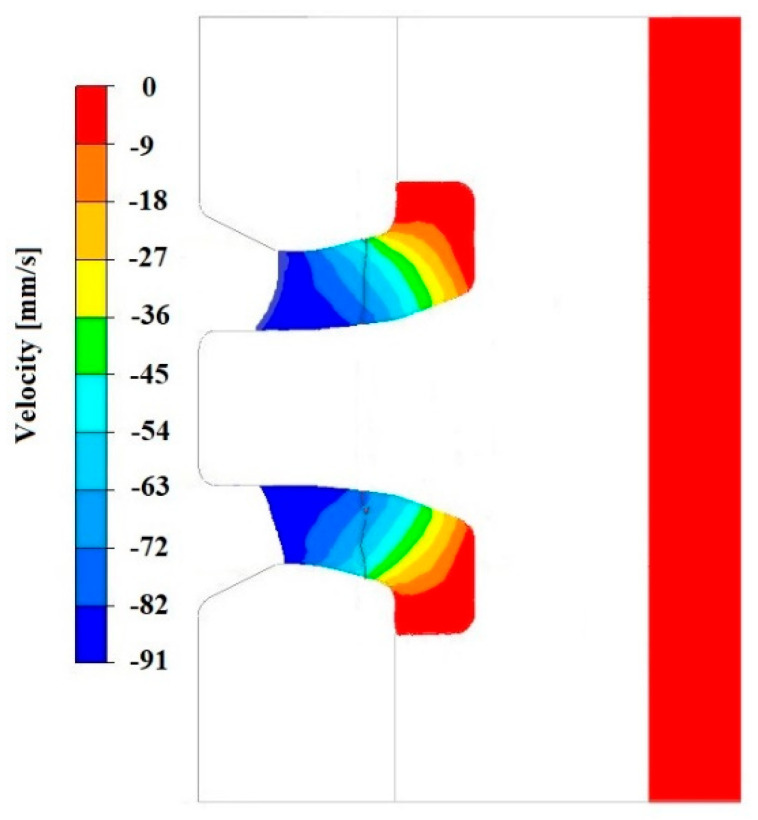
Distribution of values of material flow velocity in the longitudinal section of the welded pipe, with a calibrating die section length of 1 mm, a welding chamber length of 2 mm, and a mandrel apex angle in the welding chamber of 21°.

Figure 19, Figure 20, Figure 21, Figure 22, Figure 23 and Figure 24 show example distributions of hydrostatic pressure values and material flow velocity in the longitudinal section of the welded pipe. For these examples, a constant length of the calibrating section equal to 1 mm and a welding chamber length of 2 mm were assumed. The variable parameter was the mandrel apex angle in the welding chamber, with values in the range of 21–41°. Negative values of metal flow velocity result from the fact that the movement of the punch occurs in the direction opposite to the adopted **x**-axis orientation.

Based on the data presented in Figure 20, Figure 22 and Figure 24, it can be stated that the uneven distribution of material flow velocity in the welding chamber results primarily from the geometry of the tool. Changing the value of the mandrel generatrix angle affects the direction and nature of material flow. With a larger mandrel generatrix angle, the volume of the welding chamber decreases, which leads to an increase in material flow velocity, especially in the region near the outer diameter of the tube.

During the extrusion process, the material flows through the welding chamber, bypassing the mandrel, which causes the formation of an uneven velocity field. In the region near the outer diameter of the tube, the material has more space to flow, frictional resistance is lower due to the absence of the mandrel, and the metal flow is more stabilised, which favours higher flow velocity values. In contrast, in the region near the inner diameter, close to the mandrel, the material flow is restricted. Here, streams from different parts of the cross-section converge and combine, causing an increase in compressive stresses and disturbances in the velocity field, which consequently reduces the flow velocity values.

The variation in material flow velocity directly affects the distribution of hydrostatic pressure in the welding chamber. The lowest pressure values occur in the region near the outer diameter of the tube, where the metal flow is freer and faster, whereas the highest hydrostatic pressure values appear in the region near the inner diameter, close to the mandrel, where the material flow is restricted and higher compressive stresses occur (Figure 19, Figure 21 and Figure 23).

**Figure 25:**
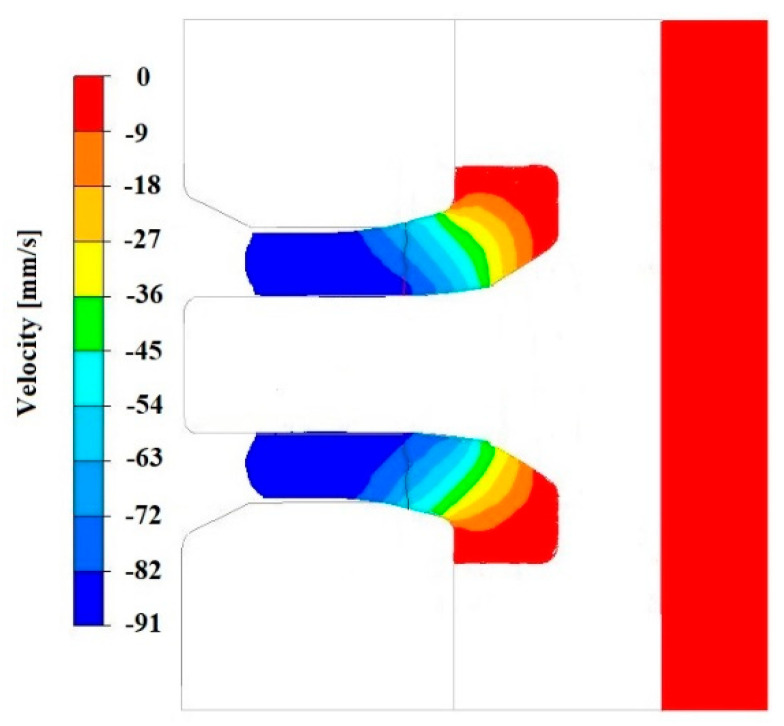
Distribution of values of material flow velocity in the longitudinal section of the welded pipe, with a calibrating die section length of 4 mm, a welding chamber length of 3 mm, and a mandrel apex angle in the welding chamber of 32°.

**Figure 26:**
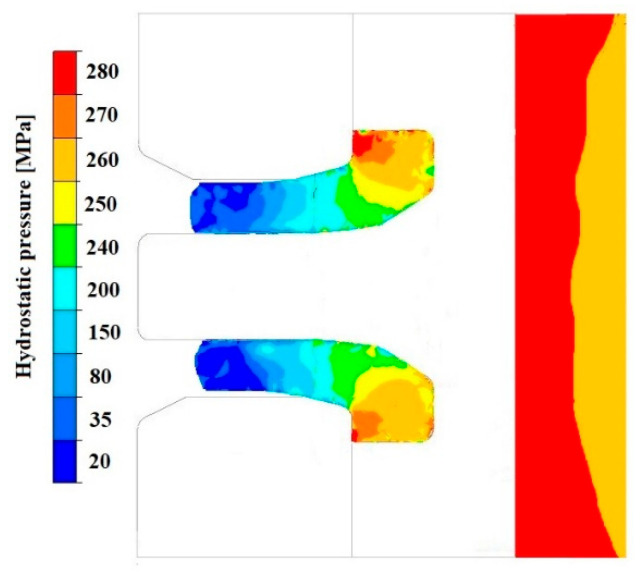
Distribution of values of hydrostatic pressure in the longitudinal section of the welded pipe, with a calibrating die section length of 4 mm, a welding chamber length of 3 mm, and a mandrel apex angle in the welding chamber of 32°.

Based on the analysis of the numerical modelling results of the tube extrusion process, it was determined that the optimal geometric tool parameters favouring the welding process are a calibration section length of 4 mm, a welding chamber length of 3 mm, and a mandrel angle in the welding chamber of 32°. For this case, uniform material flow in the welding chamber was achieved, which results in a consistent wall thickness around the circumference of the pipe (Figure 25). For these parameters, the hydrostatic pressure values were significantly higher than those required to achieve a permanent joint of oxygen-free copper in the welding process (Figure 26), as confirmed in the physical simulation of pressure welding.

## 5. Conclusions

Based on the analysis of the research results, the following final conclusions were formulated:

1. Tool geometry: the mandrel angle in the welding chamber, the length of the welding chamber, and the length of the calibration section of the die have a significant impact on the material flow kinematics in the tube welding process.
2. Increasing the mandrel angle in the welding chamber leads to higher hydrostatic pressure values in the deformation cavity.
3. Extending the welding chamber improves welding conditions by increasing hydrostatic pressure; however, excessive extension increases frictional resistance and hinders material flow.
4. The length of the die calibration section significantly affects welding conditions—its extension increases hydrostatic pressure and promotes welding, but at the same time raises flow resistance and reduces extrusion efficiency.
5. The minimum geometric parameters of the die required to achieve a durable joint are mandrel angle in the welding chamber—21°, length of the welding chamber—2 mm, and length of the calibration section of the die—1 mm.
6. The results of the numerical simulations were confirmed by physical experiments.

Both numerical analyses and experimental studies confirmed the significant influence of tool geometry on the distribution of hydrostatic pressure and the material flow characteristics in the extrusion of oxygen-free copper pipes. Proper selection of the structural parameters of the welding chamber and the calibration section facilitates hydrostatic pressure values sufficient to ensure a durable joint in the oxygen-free copper pipe welding process.

The optimal geometric parameters of the tools are a length of the calibration section of the die of 4 mm, a length of the welding chamber of 3 mm, and a mandrel angle in the welding chamber a 32°. For these parameters, the difference in hydrostatic pressure between the inner and outer surfaces of the tube is minimised, and pressure in the copper joining area is intensified.

## Data Availability

The original contributions presented in this study are included in the article. Further inquiries can be directed to the corresponding author.
